# Case Report: Confocal microscopy in the early diagnosis of microsporidial keratitis

**DOI:** 10.3389/fmed.2025.1745070

**Published:** 2026-01-15

**Authors:** Jingjing Su, Ke Liu, Xiaofang Wu, Baotao Lin, Fangwei Ying, Yingting Zhu, Ming Li, Ping Guo

**Affiliations:** 1 Shenzhen Eye Hospital, Shenzhen Eye Medical Center, Southern Medical University, Shenzhen, China; 2 BioTissue, Miami, FL, United States

**Keywords:** corneal perforation, *in vivo* confocal microscopy, metagenomic sequencing, microsporidial stromal keratitis, *Vittaforma corneae*

## Abstract

This report describes a rare case of microsporidial stromal keratitis (MSK) complicated by corneal perforation in a 69-year-old male farmer with a 5-month history of ocular redness, pain, photophobia, and epiphora. *In vivo* confocal microscopy (IVCM) revealed pathognomonic findings—hyperreflective double-walled spore casings and vesicular clusters, providing the earliest diagnostic clues for microsporidia infection. Subsequent metagenomic next-generation sequencing (mNGS) and histopathology confirmed *Microsporidia* species. The patient underwent therapeutic penetrating keratoplasty followed by targeted anti-microsporidial therapy, achieving globe preservation and visual improvement. This case underscores IVCM’s pivotal role in diagnosing MSK, particularly in atypical presentations. Because MSK remains a rare corneal disorder, its insidious progression necessitates high clinical vigilance. In summary, IVCM’s ability to detect microsporidial structures in real-time significantly enhances early diagnosis, complementing molecular methods like mNGS. We conclude that IVCM, as a non-invasive and rapid diagnostic tool, provides a convenient and efficient means for the early differentiation of challenging corneal infections.

## Introduction

Microsporidial stromal keratitis (MSK) is an uncommon but vision-threatening corneal infection caused by obligate intracellular microsporidia (1). While systemic microsporidiosis was previously thought to be associated with immunocompromised patients, recent reports indicate an increasing incidence among immunocompetent individuals, particularly agricultural workers exposed to soil and water contamination (2, 3). Microsporidial keratitis was first reported in 1990 in association with *human immunodeficiency virus* (HIV) infection (4). Recent studies have also documented cases of microsporidial keratitis in immunocompetent patients (5–7). The diagnostic challenge stems from non-specific early manifestations, such as ocular redness, photophobia, and pain, which frequently mimic bacterial, fungal, viral, or even *Acanthamoeba* keratitis, often resulting in delayed intervention (8). However, current diagnostic paradigms face significant limitations: transmission electron microscopy (TEM) (9), while considered the gold standard, proves impractical for initial screening due to stringent sample requirements and relatively low sensitivity (10). Conventional light microscopy remains operator-dependent, requiring expert interpretation while lacking species-specific identification capability. Neither histopathology nor PCR testing offers widespread accessibility due to technical and cost constraints. In this context, *in vivo* confocal microscopy (IVCM) has emerged as an early, non-invasive, and rapid diagnostic tool, enabling real-time visualization of pathognomonic corneal changes. However, documented applications of IVCM in MSK remain scarce in contemporary literature (8). Herein, we present a diagnostically challenging case of MSK progressing to corneal perforation, where IVCM provides critical early diagnostic clues subsequently confirmed by metagenomic next-generation sequencing (mNGS). This report highlights (1) the indispensable role of IVCM in atypical keratitis evaluation and (2) the critical need for prompt multimodal diagnostic approaches to prevent vision-threatening complications.

## Disease presentation and ocular features

**FIGURE 1:**
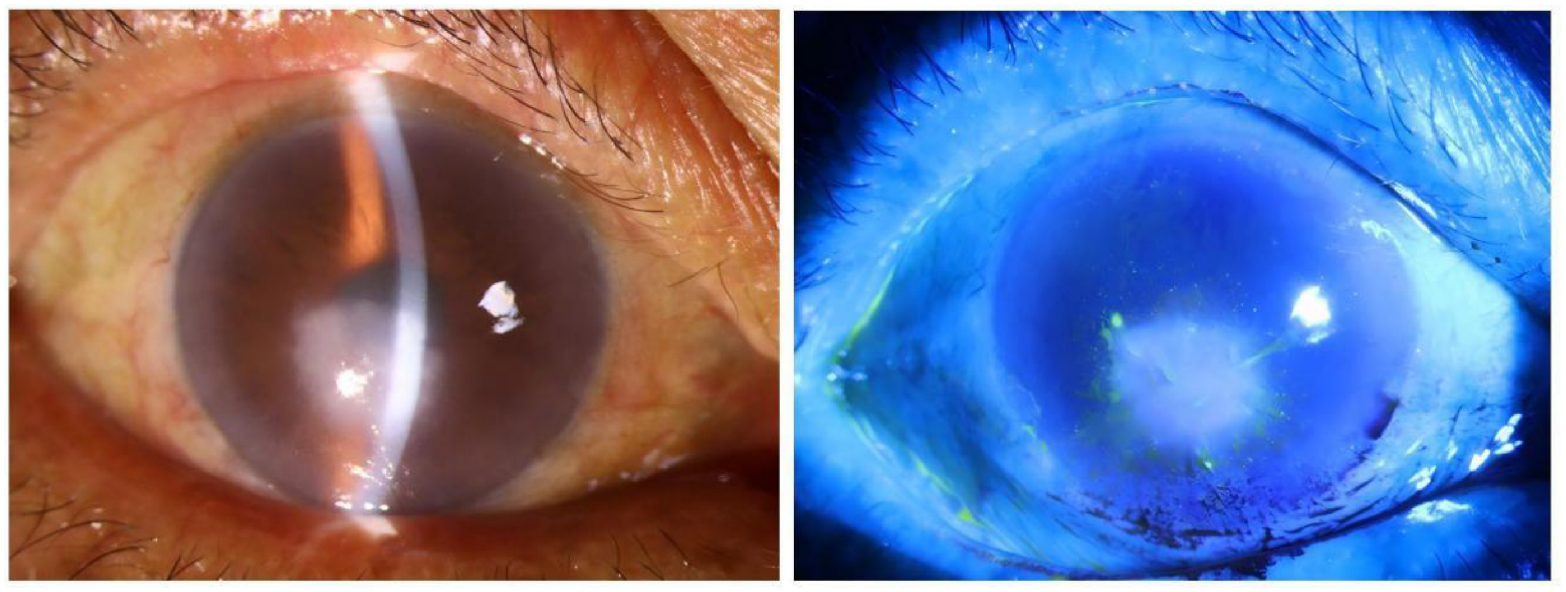
Slit-lamp microscopy examination revealed keratitis in its early stage, and fluorescein staining was negative for ulceration.

**FIGURE 2:**
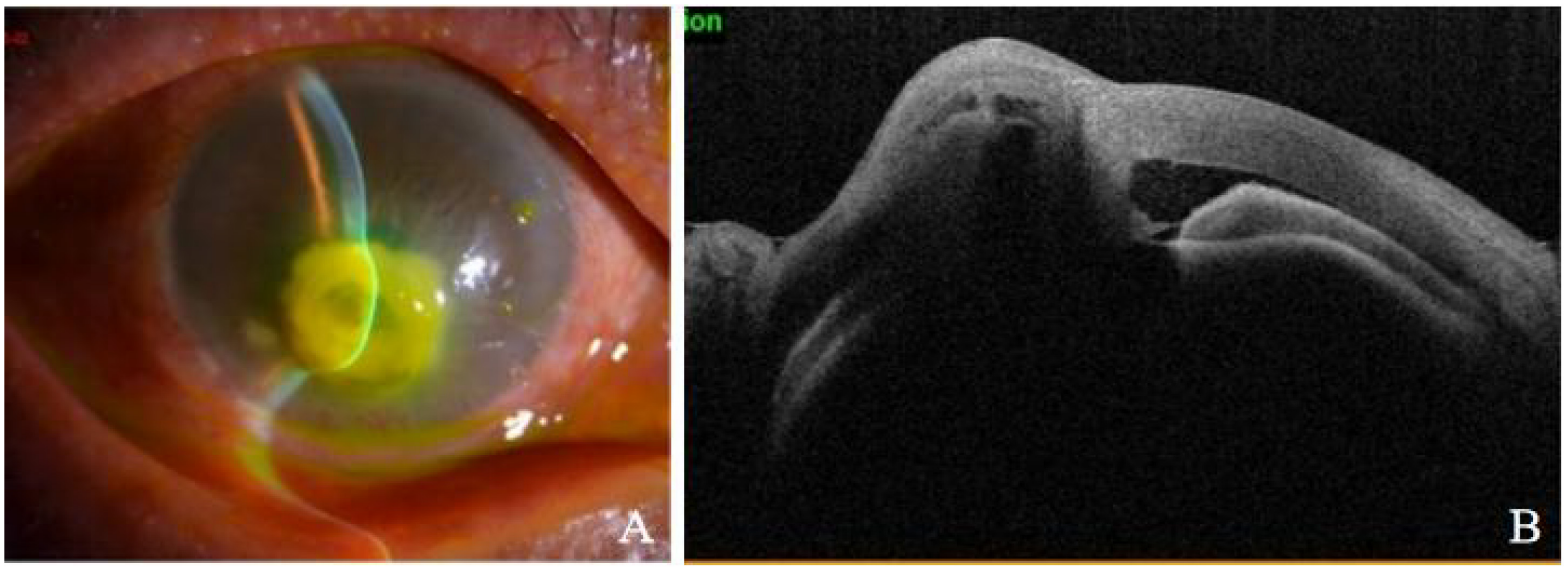
**(A)** Slit lamp imaging shows corneal ulcer perforation accompanied by iris incarceration. **(B)** The optical coherence tomography indicated the disappearance of the peripheral anterior chamber, with corneal ulcer perforation accompanied by iris tissue incarceration.

The patient was a 69-year-old male farmer who was admitted to the hospital due to redness and pain in the right eye for over 5 months, accompanied by tearing with warmth sensation for 1 week. Five months earlier, the patient developed unexplained redness and pain in the right eye, accompanied by decreased vision without increased discharge. The patient-provided eye photos revealed the following findings in the right eye: a 4 mm × 5 mm grayish-white opacity was visible inferocentral to the corneal center, with blurred margins and stromal edema. There was inferior corneal neovascularization. Keratic precipitates (KP) were present on the endothelium. The anterior chamber was clear. The pupil was round and reactive to light. Lens opacity was noted (Figure 1). The treatment regimen at the external hospital had been initially antifungal therapy, followed by antibiotic therapy, and was later changed to antiviral therapy combined with tobramycin-dexamethasone eye drops (Antifungal drugs and antibiotics were prescribed orally to the patient, but the specific types are unknown). However, the symptoms had gradually worsened. One week before admission, he developed hot tears and was diagnosed with “corneal ulcerative perforation (OD),” the initial examination revealed visual acuity of hand motion and intraocular pressure of Tn-1 in that eye. The right eye exhibited severe mixed conjunctival congestion. A dense white corneal opacity measuring approximately 6 mm × 5 mm was observed in the inferocentral cornea, containing a 5 mm × 4 mm ulcerative lesion with central perforation and iris incarceration. Purulent exudate was adherent to the lesion, accompanied by stromal edema in the peripheral cornea and radial folds in Descemet’s membrane. Corneal neovascularization was noted at the limbus, particularly prominent inferiorly. The anterior chamber depth was extremely shallow superiorly and virtually absent inferiorly. Lens opacity was present without obvious hypopyon. The pupil was measured about 3 mm in diameter with sluggish light reflex, and other intraocular structures were not clearly visible, including the fundus (Figure 2). The patient was otherwise healthy with no immunodeficiencies. In 2016, he had been successfully treated for “fungal corneal ulcer (OD)” with antifungal therapy.

## Diagnostic assessment and therapeutic intervention

**FIGURE 3:**
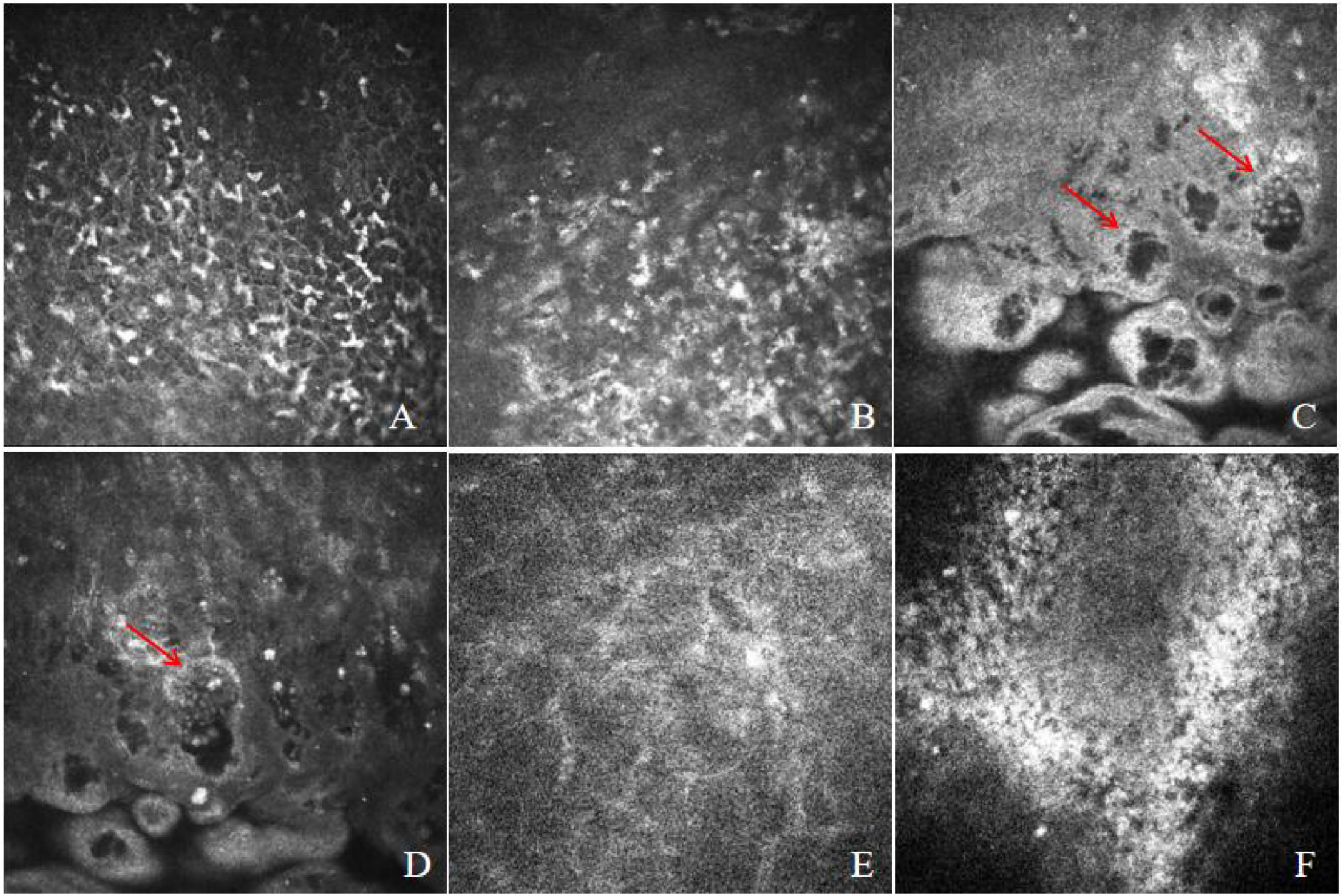
Confocal microscopy of the patient revealed numerous Langerhans cells and inflammatory cell infiltration in the epithelial layer **(A,B)**. Multiple cyst-like structures with bright walls were observed **(C,D)**, some containing clusters of round bright spots within the wall sections, a characteristic finding of microsporidia (red arrows). **(E)** Demonstrated swollen stromal layers, while **(F)** showed extensive inflammatory cell infiltration at the endothelial surface.

The corneal confocal microscopy obtained on the day of admission revealed numerous Langerhans cells and round inflammatory cell infiltrates in the corneal epithelium. Multiple cyst-like structures with bright walls were observed, some of which contained clustered-round bright spots within the wall sections, suggesting microsporidial infection (Figure 3). The stromal cells exhibited significant swelling with unclear imaging, and some cross-sections showed abundant inflammatory cell infiltrates in the endothelial cells.

**FIGURE 4:**
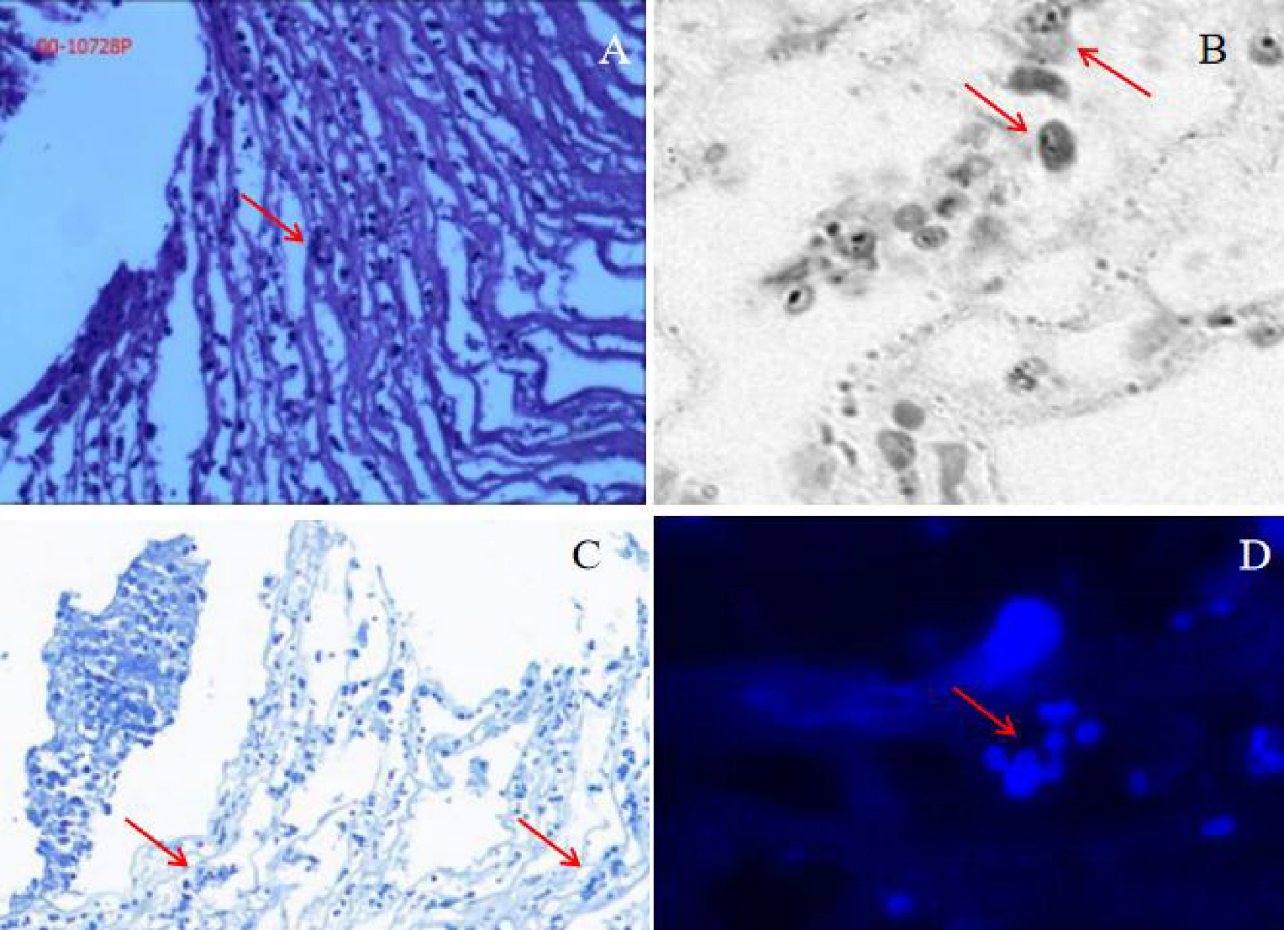
Diagnostic microscopy and staining of microsporidia (red arrows). **(A)** Pathological section. **(B)** Ink-stained pathological section. **(C)** Toluidine blue staining. **(D)** Calcofluor White M2R fluorescent staining. Arrows indicate strongly fluorescent blue microsporidial structures.

Based on the IVCM results, the team initiated a regimen of frequent topical voriconazole and natamycin eye drops, supplemented with levofloxacin eye drops for antibacterial therapy, along with systemic intravenous voriconazole. To salvage the globe, a penetrating keratoplasty was arranged and performed on the following day. Intraoperatively, corneal tissue samples were obtained and subjected to metagenomic sequencing and pathological examination. The metagenomic results indicated that the patient was infected with *Vittaforma corneae* (Table 1). Furthermore, microsporidial structures were identified through Calcofluor White M2R staining of corneal lesion sections (Figure 4).

**TABLE 1:** The results of metagenomics next-generation sequencing.

Category	Species	Name	Sequences
Eukaryota	Vittaforma	*Vittaforma corneae*	22,430
Bacteria	Cutibacterium	*Cutibacterium acnes*	3
Viruses	Lymphocryptovirus	*Human gammaherpesvirus 4*	2
Bacteria	Acinetobacter	*Acinetobacter johnsonii*	1
Bacteria	Corynebacterium	*Corynebacterium matruchotii*	1

## Follow up and outcomes

**FIGURE 5:**
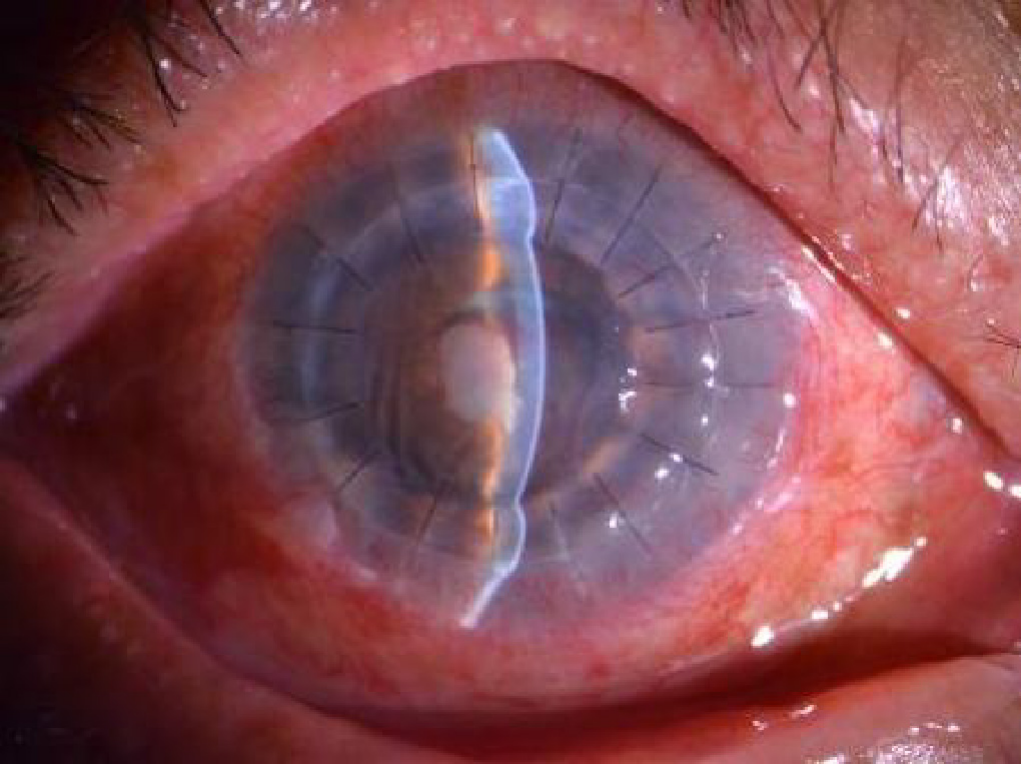
Anterior segment photograph taken 1 month later.

Postoperatively, the patient was managed with a regimen of voriconazole eye drops (every 2 h), natamycin eye drops (every 2 h), levofloxacin eye drops (four times daily), ofloxacin eye ointment combined with fluconazole eye ointment (once nightly), and tacrolimus eye drops (once daily). Two weeks later, the corneal graft stabilized, allowing for a gradual reduction in the antifungal medications. Concurrently, steroid eye drops were introduced and the frequency of tacrolimus was increased to four times daily. One month postoperatively, the patient’s condition improved, with the corneal graft remaining transparent and without any signs of recurrence. Subsequent cataract surgery was recommended to improve visual acuity (Figure 5). Due to the significant distance from the hospital, the patient was unable to return for in-person follow-up. During a telephone follow-up, the patient reported no discomfort. Currently, the patient continues anti-rejection medication. However, long-term complication risks cannot be effectively monitored.

## Discussion

Microsporidia are obligated intracellular parasitic fungi with a highly simplified genomic structure. They lack mitochondria and invade host cells through polar tubes, forming a specific symbiotic relationship with the host (11). Microsporidial keratoconjunctivitis (MKC) is primarily caused by *Enterocytozoon*, whereas microsporidial stromal keratitis (MSK) is mainly attributed to *Vittaforma corneae*. Clinically, MKC is commoner, while MSK is rare (1, 12–15).

Common symptoms of MKC include eye redness, tearing, foreign body sensation, and varying degrees of visual impairment. Ocular signs are often present as multifocal, superficial punctate, or roundish epithelial lesions, which are slightly elevated with a rough surface, and may or may not be accompanied by superficial stromal infiltration. Conjunctival manifestations may include follicular reaction or pseudomembrane formation (1, 12–14). MSK is a rare form characterized by insidious onset, prolonged course, long latency, high recurrence rate, and poor prognosis. Its symptoms are non-specific, often including eye redness, pain, photophobia, foreign body sensation, and decreased vision. Signs predominantly include diffuse or multifocal stromal granular or crystalline infiltrates, possibly accompanied by mild to moderate conjunctival hyperemia, stromal edema, or endothelial deposits. The corneal epithelium is mostly intact and rarely defective (1, 12–14, 16).

Microsporidial keratoconjunctivitis and MSK present with diverse clinical manifestations, posing significant diagnostic challenges as they can be easily confused with multiple other conditions. MKC is often mistaken for viral keratitis, Thygeson’s superficial punctate keratitis (TSPK), and epidemic keratoconjunctivitis. Furthermore, due to its insidious onset, MSK is frequently misdiagnosed as *Acanthamoeba* keratitis (AK), herpes simplex virus keratitis, or fungal keratitis (1, 12–14, 16).

Among these, adenoviral keratoconjunctivitis typically presents as a bilateral infection characterized by multifocal, fine punctate subepithelial infiltrates (17). In contrast, TSPK manifests as bilateral, asymmetrical, coarse, oval, and slightly elevated granular epithelial or subepithelial lesions that predominantly affect the central cornea while sparing the periphery, with the underlying stroma and conjunctiva generally remaining unaffected (18, 19). HSK is traditionally classified by its primary anatomical site of involvement into epithelial, stromal, endothelial, neurotrophic, or mixed forms, commonly presenting with signs such as corneal infiltration, opacity, edema, folds in Descemet’s membrane, and posterior corneal deposits (20). The presence of an irregular/feathery border, satellite lesions, and endothelial plaque is associated with fungal keratitis (21). Meanwhile, the presence of radial keratoneuritis, an early characteristic manifestation presented as radial, linear, and branching stromal infiltrates extending from the central cornea should raise suspicion for *Acanthamoeba* keratitis (AK), though this finding can also be present in bacterial infections such as Pseudomonas keratitis. Strong suspicion for AK is further warranted when corneal epitheliopathy, endothelial plaque, radial keratoneuritis, and annular infiltration occur together, especially in cases unresponsive to standard antibacterial or antiviral therapy (22–24). Given the substantial overlap in clinical features among these entities, enhancing capabilities for differential diagnosis and laboratory testing is crucial. In this case as shown in Figure 1, the clinical presentation of microsporidial stromal keratitis (MSK) closely resembled that of herpes simplex virus keratitis (HSK). Both conditions exhibited corneal stromal haze, edema, and inflammatory keratic precipitates (KP) on the endothelial surface. However, after treatment with a combination of antiviral agents and corticosteroids, the conditions showed no improvement and ultimately progressed to corneal ulceration and perforation.

A retrospective study systematically analyzed the etiology, pathogenic microorganisms, risk factors, treatment, and prognosis of nearly 2,000 cases of infectious keratitis (IK) at the National Eye Hospital of Vietnam. Although half of the cases were successfully treated with medications alone, the medication treatment failure rate was highest for microsporidial keratitis (38.2%) followed by fungal keratitis (28.1%) (25). Early diagnosis and early treatment are particularly important for microsporidial keratitis.

Currently, the diagnosis of microsporidial keratoconjunctivitis largely relies on clinical presentation supplemented by microbiological analysis of corneal scrapings and PCR testing. Traditional staining and culture methods have significant limitations in sensitivity, often leading to misdiagnosis (26). With advances in molecular biology, newer techniques such as PCR and high-throughput sequencing have significantly improved detection sensitivity and specificity, facilitating early pathogen identification.

Numerous studies underscore the importance of PCR as an essential tool for improving the detection of these rare conditions, addressing the limitations of standard diagnostic approaches (27, 28). However, high costs and time-consuming procedures hinder rapid confirmation (1, 13, 27, 29).

*In vivo* confocal microscopy plays an increasingly vital role in the early diagnosis of infectious keratitis. Current evidence supports its value not only in detecting *Acanthamoeba* keratitis (AK) and fungal keratitis (30), but also as highlighted in this study, in the identification of microsporidial keratitis (MKC). In AK, IVCM consistently visualizes characteristic forms: cysts (12–25 μm, with a hyporeflective wall and a bright, irregularly-shaped core) and trophozoites (20–60 μm, hyperreflective, amorphous) (23, 31). Similarly, in fungal keratitis, it clearly reveals fungal hyphae as linear, hyper reflective, branching structures approximately 3–8 μm in width, sometimes resembling double-walled filaments (31).

This study shows the cyst wall and vesicular structures of microsporidia in living tissue. IVCM is rapid, non-invasive and high-resolution, making it suitable for auxiliary diagnosis, treatment monitoring, and follow-up evaluation of microsporidial keratitis (32). Previous diagnoses often relied on observing rosette-like clusters of epithelial cells containing highly reflective needle-shaped or oval bodies, yet these were difficult to distinguish definitively from inflammatory cells, amoebic cysts, or fungal structures (1, 12, 14, 28). This case successfully captured diagnostic vesicular structures of microsporidia in deep corneal stroma via IVCM, filling a gap in the application of IVCM for auxiliary diagnosis of MKC. The images clearly show microsporidial vesicles measuring approximately 15–25 μm in diameter, containing highly reflective spores (about 2–4 μm), with distinctly hyperreflective cyst walls. This finding will significantly enhance the clinical diagnosis of microsporidial keratitis. It is crucial to emphasize that the diagnostic yield of this examination is critically dependent on the operator’s expertise. Accurate identification of its subtle signs and a consequent increase in diagnostic value require a physician with a profound understanding of microsporidial morphology and proficient instrumental skills.

Microsporidial keratitis is a rare yet clinically challenging ocular infection, the accurate diagnosis of which relies heavily on a high index of clinical suspicion and comprehensive diagnostic integration. In this case, the disease was systematically characterized through a multimodal approach incorporating *in vivo* confocal microscopy (IVCM), metagenomic testing, pathological staining, and enhanced staining techniques. Notably, the successful capture of typical microsporidial vesicular structures under IVCM provided crucial evidence for early diagnosis. This breakthrough overcomes the limitations of conventional diagnostic methods and opens new avenues for improving diagnostic precision and therapeutic outcomes.

Furthermore, the progression of keratitis to corneal ulceration and perforation in this case holds considerable instructional value. The distinctive microsporidial morphology visualized by confocal microscopy not only served as a key diagnostic clue but also enhanced clinical recognition of the disease. These insights are expected to facilitate the early detection of suspected microsporidial keratitis in the future, thereby reducing the risk of misdiagnosis and inappropriate medication use attributable to insufficient disease awareness.

## Data Availability

The original contributions presented in this study are included in this article/supplementary material, further inquiries can be directed to the corresponding authors.
